# Report on Military Prisons

**Published:** 1857-01

**Authors:** 


### REPORT ON MILITARY PRISONS.

Lieutenant-Col. Jebb has brought out a report, which is satisfactory, in respect to the health of military prisoners. Since 1850, a reduced diet has rather improved the health of the men than otherwise. Here is the scale:—

| | *Ordinary.* | | | *After* 84 *Days.* | |
|---|---|---|---|---|---|
| Breakfast | Oatmeal .... | 8 oz. | .... | Oatmeal .... | 10 oz. |
| Dinner .. | Indian meal . | 9 | .... | Indian meal . | 12 |
| Supper .. | Bread ...... | 8 | .... | Bread ...... | 8 |

With half a pint of milk to each meal. This diet leads to loss of bodily weight, but to less sickness. Like all these prison documents and modern preachments about model plans of treating prisoners, this Report is hopelessly superficial and unnatural. If prison-reform, so called, has to rest on "model discipline," so called, then Heaven, or a revolver, protect us from liberated prisoners. We do not often argue in poetry, but Wordsworth has a song so moulded to our hands, that we use it without hesitation:—

"This is the process of our love and wisdom!
To each poor brother who offends against us,
Most innocent, perhaps,—and what if guilty?
Is this the only cure?
. . . . . . '*Uncomforted*
*And friendless solitude!*' . . .
With other ministrations thou, oh Nature!
Healest thy wandering and distempered child.
Thou pourest on him thy soft influences,
Thy sunny hues, fair forms, and, breathing sweets,
Thy melodies of woods and winds and waters,
Till he relent, and can no more endure
To be a jarring and dissonant thing
Amid this general dance and minstrelsy;
But, bursting into tears, wins back his way,
His angry spirit healed and harmonized
By the benignant touch of love and beauty."

